# The association of fibrinogen–albumin ratio and neutrophil–lymphocyte ratio with the severity of respiratory syncytial virus infection in children

**DOI:** 10.1590/S1678-9946202466026

**Published:** 2024-04-19

**Authors:** Zeynep Uze Okay, Berker Okay, Halil Ugur Hatipoglu, Gulsen Akkoc, Kamil Sahin

**Affiliations:** 1 University of Health Sciences, Haseki Training and Research Hospital, Department of Pediatrics, Sultangazi, Istanbul, Turkey; 2 Marmara University, Pendik Training and Research Hospital, Department of Pediatric Infectious Diseases, Pendik, Istanbul, Turkey

**Keywords:** Lower respiratory tract infection, Children, Fibrinogen–albumin ratio, Neutrophil–lymphocyte ratio, Respiratory syncytial virus

## Abstract

Respiratory syncytial virus (RSV) is a common cause of respiratory infections. It is responsible for more than half of lower respiratory tract infections in infants requiring hospitalization. This study aimed to investigate the correlation between the fibrinogen–albumin ratio (FAR) and the severity of RSV infection and to compare its effectiveness with the neutrophil–lymphocyte ratio (NLR). This was a retrospective cohort study with patients aged from 29 days to two years who had been admitted to the pediatric clinic of our hospital. Patients were divided into four groups: group 1 (mild disease), group 2 (moderate disease), group 3 (severe disease), and group 4 (control). FAR and NLR were measured in all groups. FAR was significantly higher in group 3 than in the other groups, in group 2 than in groups 1 and 4, and in group 1 than in group 4 (p<0.001 for all). NLR was significantly higher in group 4 than in the other groups and in group 3 than in groups 1 and 2 (p<0.001 for all). FAR totaled 0.078 ± 0.013 in patients with bronchiolitis; 0.099 ± 0.028, in patients with bronchopneumonia; and 0.126 ± 0.036, in patients with lobar pneumonia, all with statistically significant differences (p<0.001). NLR showed no significant statistical differences. This study found a statistically significant increase in FAR in the group receiving invasive support when compared to that receiving non-invasive support (0.189 ± 0.046 vs. 0.112 ± 0.030; p=0.003). Mechanical ventilation groups showed no differences for NLR. FAR was used to identify severe RSV-positive patients, with a sensitivity of 84.4%, a specificity of 82.2%, and a cutoff value of >0.068. This study determined a cutoff value of ≤1.49 for NLR, with a sensitivity of 62.2% and a specificity of 62.2% to find severe RSV-positive patients. Also, statistically significant associations were found between FAR and hospitalization and treatment length and time up to clinical improvement (p<0.001 for all). NLR and hospitalization and treatment length showed a weak association (p<0.001). In children with RSV infection, FAR could serve to determine disease severity and prognosis and average lengths of hospitalization, treatment, and clinical improvement. Additionally, FAR predicted disease severity more efficiently than NLR.

## INTRODUCTION

Lower respiratory tract infections (LRTIs) constitute an important cause of mortality and morbidity in childhood^1^. Viral agents configure the leading cause (50–60%) of LRTIs among children under the age of 5 years; a rate that increases to 80% at under two years^2^. Among the viruses that cause LRTIs, respiratory syncytial virus (RSV) is responsible for more than half of the cases requiring hospitalization in young children^2^.

Fibrinogen and albumin are plasma proteins synthesized in the liver^3,4^. Several studies have shown an elevated fibrinogen–albumin ratio (FAR) in conditions such as coronary artery disease, stroke, rheumatoid arthritis, and COVID-19, with a relationship to the severity and mortality of the disease^5^. The neutrophil–lymphocyte ratio (NLR) has recently gained prominence as an inflammation marker. Neutrophil count typically increases during inflammation, whereas that of lymphocytes decreases, rendering NLR a sensitive indicator^6^. Numerous studies have employed NLR to indicate mortality and its elevation in conditions such as bacteremia, sepsis, and COVID-19, also suggesting its potential as a predictor for adverse outcomes in RSV patients^7-9^.

This study aimed to identify the relationship between FAR and the severity of RSV infection. It was conducted to guide physicians by predetermining the factors affecting the disease course and the severity of RSV infection, which has no specific treatment and might have a high mortality and morbidity depending on age and comorbidities. This study also aimed to determine the factors affecting FAR levels and to assess whether FAR showed superior predictive power over NLR.

## MATERIALS AND METHODS

Patients aged 29 days to two years who were admitted to the pediatric unit of University of Health Sciences, Istanbul Haseki Training and Research Hospital from December 1^st^, 2021 to January 1^st^, 2023, were found to be RSV direct fluorescent antibody test (DFT)-positive, and received fibrinogen and albumin tests were included in this retrospective cohort study. This study was carried out at a university hospital situated in Istanbul, Turkey. The patient cohort for this study was derived from individuals for whom the RSV-DFT assay was requested, identified by the hospital information system using the assay entry code. The sample for this study was then assembled by scrutinizing the list of patients who tested positive for RSV-DFT and those who underwent a fibrinogen-albumin assay (following the SARS-CoV-2 outbreak at our hospital, a protocol implemented by the Pediatric Infection Clinic involves requesting fibrinogen and albumin tests for all patients in whom viral agents are detected in respiratory tract swabs). Control group members were identified by the ICD code. Children with negative acute phase reactants for whom fibrinogen and albumin tests were requested were selected from patients without active complaints or chronic diseases applying to the hospital for routine tests.

**Figure 1:**
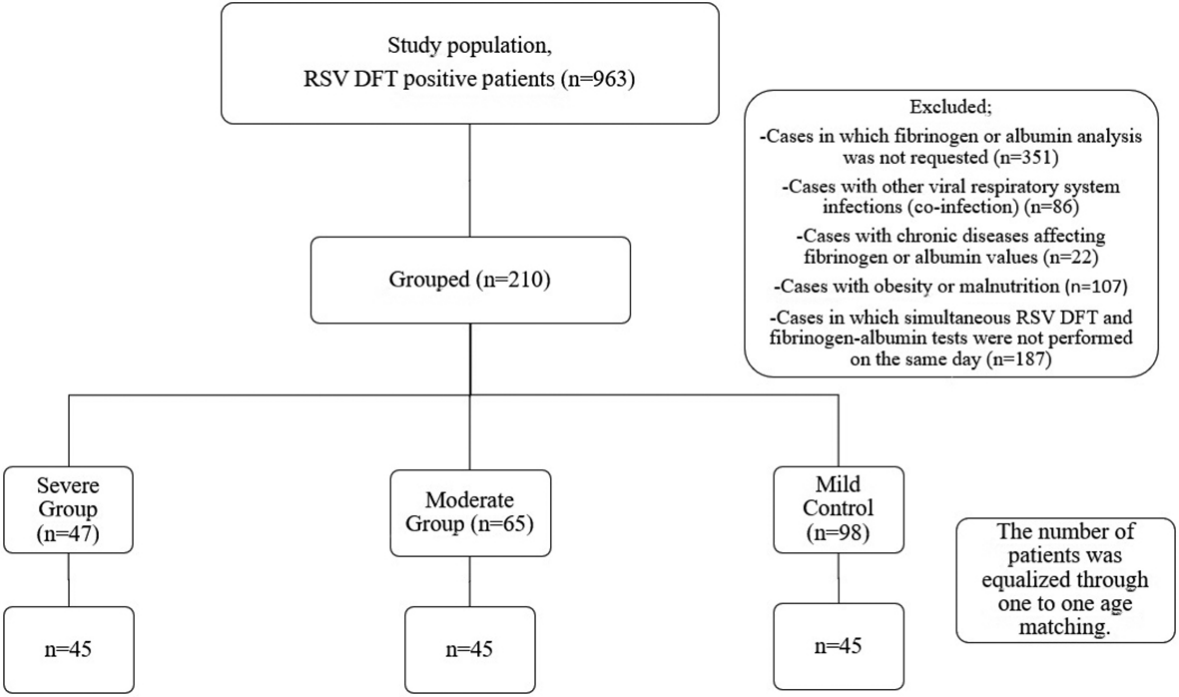
Flowchart of this study. RSV = Respiratory syncytial virus; DFT = Direct fluorescent antibody test.

The patients were divided into four groups: i) RSV DFT-positive patients who were followed up as outpatients (group 1, mild disease); ii) RSV DFT-positive patients who needed hospitalization (group 2, moderate disease); iii) RSV DFT-positive patients who needed mechanical ventilation (invasive or non-invasive; group 3, severe disease); and iv) control group patients who had no complaints, were routine controls, and were found to be RSV DFT-negative (group 4, control). A sample group was created by screening patients for each group with inclusion and exclusion criteria. The power analysis to evaluate the efficacy of FAR in indicating the severity of illness in RSV cases required 33 patients for each group. Thus, this study was structured with a 45-patient cohort, ensuring an 80% statistical power at a 0.05 significance level in a two-sided test. Power analysis was conducted to ascertain the necessary sample size to establish the association between FAR and the severity of RSV infection. Patients with other viral respiratory system infections (coinfection), chronic diseases affecting fibrinogen or albumin values (e.g., hypoalbuminemia and coagulation disorders), and a history of continuous drug use, obesity, or malnutrition were excluded. Additionally, patients without simultaneous RSV DFT and fibrinogen-albumin tests on the same day were also excluded. As a result of screening, 210 patients eligible for this study were found: 98 for group 1, 65 for group 2, and 47 for group 3. Age-matching was conducted on a one-to-one basis between groups, resulting in the selection of 45 patients for each group (Figure 1). The control group was formed with similar age and gender distributions. Patients who needed mechanical ventilation were deemed severely ill; those who needed hospitalization, moderately ill; and outpatients, as mildly ill^10^.

FAR was measured by dividing fibrinogen values (g/L) by those for albumin (g/L). NLR was obtained by dividing the neutrophil (u/L) count by the lymphocyte (u/L) count.

Time up to clinical improvement was determined as the length of time from admission to the day when dependence on inhaler therapy decreased for ward-admitted patients and from the initiation of mechanical ventilation to the day of ventilator weaning for patients under respiratory support. Treatment length was defined as the period covering the use of medication during hospitalization up to discharge. These parameters were exclusively assessed for hospitalized patients.

All chest radiographs were assessed by the same clinician, and diagnoses were established in conjunction with clinical findings. Bronchiolitis was identified in patients with clinical symptoms and flattening of the ribs and diaphragm, peribronchial thickening, and air bronchograms on the posterior-anterior chest X-ray. Pneumonia was characterized in patients with diffuse infiltration, whereas lobar pneumonia was designated for cases in which infiltrations were more localized in a single lobe or involved atelectasis.

SPSS 15.0 for Windows (SPSS Inc., Chicago, Illinois, USA) was used for statistical analyses. The Shapiro–Wilk test was used to determine whether the variables were normally distributed. Descriptive statistics were used to present the variables: numbers and percentages for categorical variables; mean, standard deviation, minimum, maximum, and median values for numerical variables. Categorical variables were compared using the chi-squared test. The Student’s *t*- and the Mann–Whitney U tests were used to compare mean or median values between groups depending on the sample distribution. Subgroup analyses were performed with the Mann–Whitney U test and interpreted with the Bonferroni correction. If both parameters were normally distributed, their correlation coefficients and significance were calculated using Pearson’s test. Otherwise, the Spearman’s test was used. Independent comparisons of more than two groups were made by one-way analysis of variance (ANOVA) for normally distributed numerical variables and the Kruskal–Wallis test for non-normally distributed variables. Statistical significance was set at p<0.05. Post hoc analysis was used to understand the statistical difference between any two groups. Factors determining the receiver operating characteristic (ROC) curve by group were analyzed by unadjusted logistic regression analysis. The capacity of FAR and NLR levels to predict a severe clinical course was analyzed using ROC curve analysis. For each parameter, the sensitivity, specificity, positive predictive value (PPV), negative predictive value (NPV), positive likelihood ratio (LR+), negative LR (LR−), accuracy, and area under the ROC curve (AUC) were calculated as diagnostic tools for predicting a moderate-to-severe clinical course. When evaluating the AUC, a 5% type-I error level was used to define a statistically significant predictive value of the test variables. Cutoff values were analyzed by ROC curve analysis.

This study was approved by the Health Sciences University Haseki Training and Research Hospital Clinical Research Ethics Committee on February 1^st^, 2023, under decision Nº 22-2023.

## RESULTS

### Demographic, clinical, and laboratory findings of RSV patients

Of the 180 patients included in the study, 91 were boys (50.5%) with median age of 4.2 (1–22) months. The most common complaints referred to cough (95.8%), wheezing (82.9%), nasal congestion (57%), and fever (35.58%). Among the study population, 77.7% of patients had pathologic examination findings in their respiratory system such as rale or rhoncus on auscultation or tachypnea.

Table 1 shows the laboratory values for the groups. When these values were analyzed based on age, neutropenia (12.2%) was observed in 22 patients; leukocytosis, in 21 (11.7%); anemia, in 17 (9.4%); and lymphopenia, in three (1.7%). Except for the frequency of leukocytosis, groups showed no statistical differences for these parameters. The frequency of leukocytosis was statistically significantly higher in group 3 compared to the other groups according to age (p=0.012).

**Table 1:** Comparison of laboratory values for the groups.

	Group 1	Group 2	Group 3	Group 4	p*

	Mean ± Standard Deviation Median (Min-Max)	Mean ± Standard Deviation Median (Min-Max)	Mean ± Standard Deviation Median (Min-Max)	Mean ± Standard Deviation Median (Min-Max)	
Leukocyte/uL	9838 ± 4401	9937 ± 3393	11508 ± 5391	9597 ± 2906	0.442
Neutrophil /uL	2370 (950-11010)	3240 (780-15320)	3980 (780-14840)	4100 (1360-10130)	**0.002**
Lymphocyte /uL	4790 (790-14270)	5160 (1110-13350)	4660 (910-14310)	3570 (1120-10100)	0.052
NLR	0.52 (0.16-2.42)	0.54 (0.07-6.96)	0.87 (0.28-15.3)	1.14 (0.2-3.85)	**<0.001**
CRP mg/L	2.1 (0.3-21.8)	2 (0.3-81.5)	19.8 (0.3-272.4)	1.3 (0.3-6.3)	**<0.001**
Fibrinogen g/L	2.78 ± 0.50	3.40 ± 0.39	4.74 ± 1.31	2.26 ± 0.49	**<0.001**
Albumin g/L	42.7 ± 3.1	42.0 ± 2.57	40.4 ± 4.85	43.0 ± 4.06	**0.003**
FAR	0.065 ± 0.011	0.081 ± 0.010	0.119 ± 0.038	0.063 ± 0.018	**<0.001**

NLR = neutrophil-lymphocyte ratio; CRP = C-reactive protein; FAR = fibrinogen-albumin ratio, *Kruskal-Wallis test.

### Factors increasing FAR and comparison of FAR with NLR

Table 2 shows the relation between symptoms, signs, and FAR. No statistical correlation was found between NLR and symptoms and signs.

**Table 2:** Relation between symptoms and signs and fibrinogen–albumin ratio.

Symptoms and signs (n=135)		Fibrinogen-albumin ratio (mean ± standard deviation)	p*
Fever	None	0.088 ± 0.035	0.562
n=48	Present	0.090 ± 0.030	
Cough	None	0.084 ± 0.027	0.682
n=130	Present	0.089 ± 0.034	
Wheezing	None	0.071 ± 0.015	**<0.001**
n=112	Present	0.094 ± 0.036	
Rale/Rhoncus	None	0.067 ± 0.012	**<0.001**
n=65	Present	0.097 ± 0.035	
Tachypnea	None	0.077 ± 0.018	**<0.001**
n=40	Present	0.101 ± 0.033	
Nasal discharge	None	0.083 ± 0.028	**0.034**
n=77	Present	0.093 ± 0.036	

^*^Mann-Whitney U test.

Comparing groups 2 and 3, hospitalization length averaged 6 (1–16) vs. 10 (2–48) days; that of treatment, seven (2–18) days vs. 10 (2–48) days; and length up to clinical improvement, three (2–7) days vs. five (2–22) days, respectively (p<0.001 for all). This study found a moderate association between FAR and hospitalization, treatment, and clinical improvement length. It also observed a weak association between NLR and hospitalization and treatment length (Table 3). The regression model conducted with age, gender, laboratory values, symptoms and findings, lung findings, and type of mechanical ventilatory support showed no correlation with FAR.

**Table 3:** Relations of fibrinogen–albumin ratio (FAR) with hospitalization, treatment, and clinical improvement lengths.

	Fibrinogen-albumin ratio		Neutrophil-lymphocyte ratio	

	r	p*	r	p*
Length of hospitalization	0.428	**<0.001**	0.271	**0.010**
Lenght of treatment	0.407	**<0.001**	0.348	**0.001**
Clinical recovery time	0.400	**<0.001**	0.021	0.137

*Spearman’s correlation.

**Figure 2:**
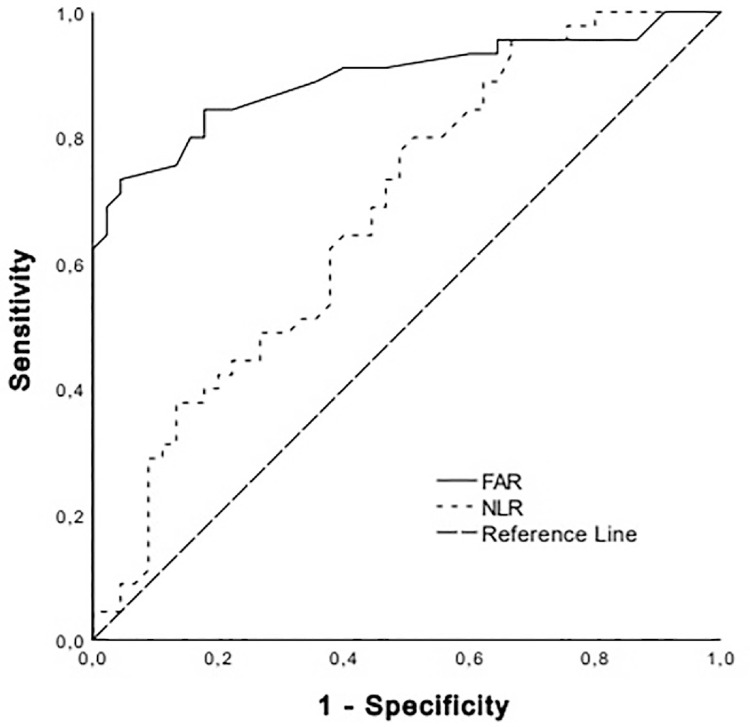
Receiver operating characteristic curve for fibrinogen–albumin ratio (FAR) and neutrophil–lymphocyte ratio (NLR) in patients with severe RSV.

Figure 2 shows the comparison of FAR and NLR values by group.

### Disease severity assessment using FAR and comparison of FAR with NLR

FAR totaled 0.078 ± 0.013 in patients with bronchiolitis, 0.099 ± 0.028 in patients with bronchopneumonia, and 0.126 ± 0.036 in patients with lobar pneumonia, all statistically significant differences (p<0.001). NLR showed no significant statistical differences [0.78 (0.54–2.76), 0.75 (0.51–2.94), and 0.81 (0.55–3.01)].

This study found a statistically significant increase in FAR in the group receiving invasive support in comparison to that receiving non-invasive support (0.189 ± 0.046 vs. 0.112 ± 0.030; p=0.003). NLR averaged 3.56 (0.58–8.95) in the group receiving non-invasive ventilatory support and 3.82 (0.62–9.03) in that receiving invasive ventilatory support, with no statistically significant differences (p=0.051).

### Comparison of the ROC curve for FAR and NLR

**Figure 3:**
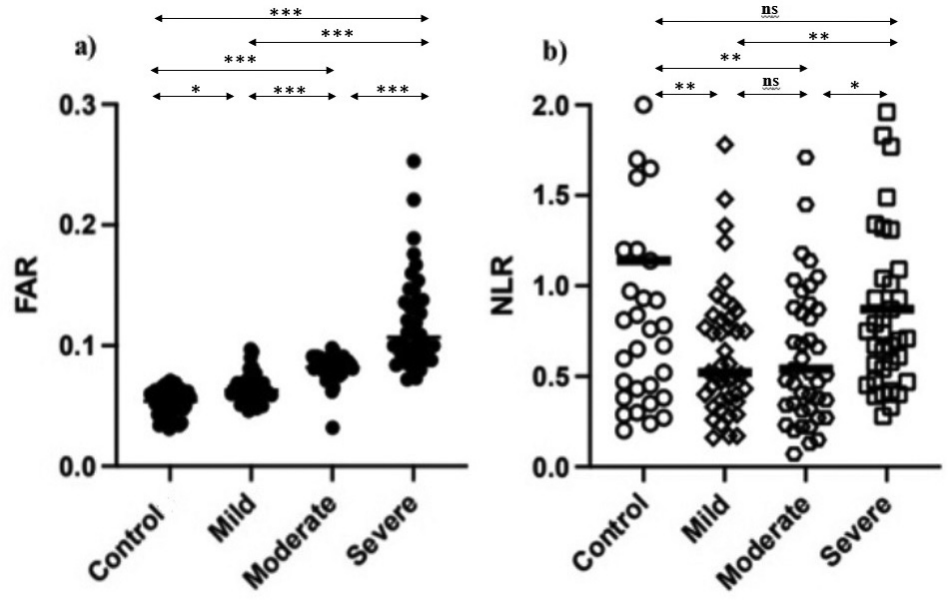
Comparison of fibrinogen–albumin (FAR) and neutrophil–lymphocyte ratios (NLR) in groups. No statistical (ns) = p≥0.05; *p<0.05; **p<0.01; ***p<0.001; FAR = fibrinogen–albumin ratio; NLR = neutrophil–lymphocyte ratio.

Figure 3 shows the ROC curve for FAR and NLR to distinguish severe RSV patients (group 3) from moderate RSV patients (group 2).

This study determined a sensitivity of 84.4% and specificity of 82.2% with a cutoff value of <0.068 for FAR in identifying RSV-positive severe patients. The FAR ROC curve analysis separated severe RSV patients from moderate ones with an AUC of 0.875 (95% CI=0.841–0.936; p<0.001). LR+ totaled 4.94 (3.18-7.68); LR−, 0.18 (0.11–0.29); PPV, 83.1% (76.0%-88.4%); NPV, 84.54% (77.2%–89.7%); and accuracy, 83.8% (77.9%–88.6%).

This study determined a cutoff value of ≤1.49 for the NLR, with a sensitivity of 62.2% and specificity of 62.2% to identify severe RSV-positive patients. The ROC curve analysis of NLR differentiated severe from moderate RSV patients with an AUC of 0.705 (95% CI=0.571–0.726; p=0.004). LR+ averaged 1.63 (1.22–2.19); LR−, 0.61 (0.46–0.82); PPV, 62% (54.8%–68.6%); NPV, 62% (54.8%–68.6%); and accuracy, 62% (54.9%–68.7%).

## DISCUSSION

The FAR was statistically significantly increased in patients with RSV infection with more symptoms and more severe pulmonary findings. FAR was higher in children with more severe diseases. We believe that measuring FAR values at admission can predict which patients will need ventilation support. Another important finding refers to the association between FAR and hospitalization, treatment, and clinical improvement length as it configured a stronger predictor than NLR.

Recently, NLR has been often used as a marker of inflammation. Neutrophil counts typically increase and those of lymphocytes decrease during inflammation, making NLR a sensitive indicator^6^. In studies conducted with healthy adults, NLR varies from 1.5 to 2.8 depending on age, gender, and genetic status^11,12^. In addition to increasing in cases such as bacteremia, sepsis, and COVID-19, some studies have used it to show mortality^7,8^. NLR can also be used to predict adverse outcomes in RSV patients.^9^ NLR has been shown to increase in patients who smoke^13^, and it may have increased in some patients in our study due to exposure to cigarette smoke. NLR was statistically and significantly higher in the ventilator-supported group than the other RSV-positive groups and was associated with disease severity.

FAR, which is the ratio between fibrinogen (an acute phase reactant) and albumina( negative phase marker) has been frequently used to predict the severity of diseases in recent years. Recent studies have shown that FAR is significantly higher in conditions such as coronary artery disease, rheumatoid arthritis, COVID-19, hepatocellular carcinoma, smoking-related endothelial damage, lung cancer, and renal cancers, and can be used to predict mortality in some cases^5,14-18^. In our study, FAR was found to indicate disease severity. Our literature review found no publication on FAR in RSV patients, and, to our knowledge, this is the first study on FAR in RSV-positive pediatric patients. Due to our retrospective and significant study, we believe that prospective and comprehensive studies on this subject may add new information.

FAR was higher in the presence of wheezing, rale or ronchus, and respiratory distress, complaints that are also related to disease severity. Again, the FAR was higher in patients with worse findings on lung imaging and a more severe prognosis. Similar to our study, FAR studies conducted with patients diagnosed with COVID-19 found that FAR can be used to predict prognoses^19-21^. In patients with difficult-to-interpret and indistinguishable lung imaging, FAR can also prevent ambiguities and enable rapid treatment planning. We believe that large-scale research is needed on this topic, including all viral LRTI agents.

Positive association existed between FAR and the lenghts of hospitalization, treatment, and clinical improvement. Similar to our study, studies conducted with COVID-19 patients have shown that FAR is associated with hospitalization and treatment length^21,22^. Measuring FAR at the time of hospital admission can determine the average length of hospital stay and treatment time in advance and prevent over- or undertreatment by planning accordingly. Additionally, since disease severity increases in subsequent days, FAR may help predict patients who might need ventilatory support at the time of admission and determine follow-up and hospitalization. In this study, FAR statistically and significantly differed in patients requiring invasive and non-invasive mechanical ventilatory support, which may help physicians during ventilatory selection in intensive care units.

New data suggest that the risk of developing recurrent wheezing or childhood asthma may be higher following an RSV infection in infancy^23,24^. Consolidating these studies, incorporating FAR, and transforming them into a comprehensive and up-to-date research initiative can offer valuable insights to the academic field. This approach can examine the relation between FAR levels in patients who had RSV in their infancy and the subsequent development or non-development of childhood asthma.

### Limitations

The limitation of this study lies in its incomplete access to some data because it was a retrospective study. We were unable to access information such as patients’ complete medical history, the time elapsed between the first symptom and the arrive to the healthcare facility, and the height and weight of patients in the outpatient and control groups. The count of identified growths in patients’ blood cultures was notably low, excluding contaminants. This limitation in data may have compromised our analysis, potentially leading to an underestimation of the impact of secondary bacterial infections on the length of hospital stay, treatment duration, and FAR levels in patients. The analyses in this study have not been adjusted for potential confounding factors due to the limited sample size within each group. The fact that this was a single-center study poses another limitation. Another notable limitation of this study is its absence of a replication cohort, particularly when describing the results obtained from the predictability analyses of FAR. FAR was evaluated once in all patients, and this study was unable to determine a correlation between the improvement in clinical conditions and the change in FAR during follow-up. Finally, another limitation refers to the inability of this study to evaluate complications and FAR in the long-term follow-up of patients after discharge.

## CONCLUSION

FAR can determine the severity and course of the disease in children with RSV infections. These easy, inexpensive, and quick tests can provide rapid diagnoses and treatment and reduce disease-related mobility and mortality rates. Early intervention and adequate supportive treatment can decrease intensive care unit admissions, complications, and hospitalization length. Additionally, the association between FAR and hospitalization, treatment, and clinical improvement length may offer easier planning and increase patient and family compliance in patients who need hospitalization. FAR predicted disease severity more accurately than NLR. Further comprehensive, multicenter, and prospective studies can increase knowledge on this subject.
